# Does Moderate Intensity Exercise Attenuate the Postprandial Lipemic and Airway Inflammatory Response to a High-Fat Meal?

**DOI:** 10.1155/2015/647952

**Published:** 2015-04-27

**Authors:** Stephanie P. Kurti, Sara K. Rosenkranz, Morton Levitt, Brooke J. Cull, Colby S. Teeman, Sam R. Emerson, Craig A. Harms

**Affiliations:** ^1^Department of Kinesiology, Kansas State University, 1A Natatorium, Manhattan, KS 66506, USA; ^2^Department of Human Nutrition, Kansas State University, Manhattan, KS 66506, USA; ^3^College of Medicine, Department of Clinical Biomedical Science, Florida Atlantic University, Boca Raton, FL 33431, USA

## Abstract

We investigated whether an acute bout of moderate intensity exercise in the postprandial period attenuates the triglyceride and airway inflammatory response to a high-fat meal (HFM) compared to remaining inactive in the postprandial period. Seventeen (11 M/6 F) physically active (≥150 min/week of moderate-vigorous physical activity (MVPA)) subjects were randomly assigned to an exercise (EX; 60% VO_2peak_) or sedentary (CON) condition after a HFM (10 kcal/kg, 63% fat). Blood analytes and airway inflammation via exhaled nitric oxide (eNO) were measured at baseline, and 2 and 4 hours after HFM. Airway inflammation was assessed with induced sputum and cell differentials at baseline and 4 hours after HFM. Triglycerides doubled in the postprandial period (~113 ± 18%, *P* < 0.05), but the increase did not differ between EX and CON. Percentage of neutrophils was increased 4 hours after HFM (~17%), but the increase did not differ between EX and CON. Exhaled nitric oxide changed nonlinearly from baseline to 2 and 4 hours after HFM (*P* < 0.05,  *η* ^2^ = 0.36). Our findings suggest that, in active individuals, an acute bout of moderate intensity exercise does not attenuate the triglyceride or airway inflammatory response to a high-fat meal.

## 1. Introduction

The prevalence of asthma has been on the rise, particularly in obese individuals [1]. Asthma is an airway hyperresponsiveness and inflammatory disorder. Specifically T helper type 2 (T_H_2) activation causes airway eosinophilia in an acquired immune response via the JAK/STAT/Raf-1 signal transduction pathway [2]. However, asthma may also be an innate immune response via MyD88/IRAK signal transduction [2]. Recent studies confirm that saturated fatty acids activate macrophages in this signal transduction pathway via nuclear factor kappa B (NF*κ*B), increasing the cytokines IL-8, IL-6, and TNF-*α* from the airway cells as well as increasing the presence of pulmonary neutrophils [3]. Fat induced activation of NF*κ*B occurs due to circulating nonesterified fatty acids (NEFA), as well as reactive oxygen species (ROS) [4]. Toll-like receptor-4 (TLR4) can also be activated by fatty acids and can activate NF*κ*B [5]. This inflammatory cascade results in an increase in inflammation in the airway via neutrophil influx and activation [6]. Ultimately, the burden of eosinophilic and neutrophilic inflammation present in asthma is reactive oxygen species (ROS) production and subsequent oxidative stress.

Typically the Western diet is calorically dense and nutrient poor, which leads to postmeal dysmetabolism and oxidative stress that increase in direct proportion to glucose and triglycerides after a meal [7]. Increased dietary fat has been found not only to activate the innate immune system, but also to increase asthma risk [8], airway hyperresponsiveness [9], and risk of chronic obstructive pulmonary disease (COPD) [10] and negatively affect asthma outcomes [11]. In the past decade, asthma prevalence has increased in Westernized countries, with over 10% of US adults diagnosed with asthma [12], which suggests that the consumption of dietary fat may play a substantial role in asthma pathology [13].

Even a single high-fat meal (HFM) leads to an increase in both systemic and airway inflammation that is thought to be associated with elevated triglycerides [14, 15]. In 2010, Rosenkranz et al. showed that airway inflammation (assessed via exhaled nitric oxide (eNO)) increased by ~20% in healthy individuals after a HFM [15]. Exhaled NO has been validated as an acceptable proxy for measuring airway inflammation [16]. Similarly, Wood and colleagues reported an increase in toll-like receptor-4 (TLR4) mRNA activation and subsequent neutrophil influx in healthy subjects in the postprandial period [17]. Neutrophil influx and activation have been linked to asthma development and also endothelial dysfunction [18]. Clearly previous research supports the notion that the increased airway inflammation is associated with an increased postprandial lipemia (PPL).

Previous work aimed at modulating postprandial airway inflammation has focused on modification of fat quality [17] or using dietary supplementation [19]. Ade and colleagues reported that, after three weeks of fish oil supplementation in the experimental group, there was no change in eNO after HFM when compared to the control group (~20% increase in eNO after HFM). Researchers hypothesized that the experimental group had a decreased sensitivity to triglycerides after the supplementation. Therefore it is important to explore other methods to modulate airway inflammation and diminish the potential effects of triglycerides on the airways. To our knowledge, no research has investigated how an acute bout of exercise or physical activity status affects the postprandial airway inflammatory response.

Chronic exercise training has been shown to reduce basal levels of systemic inflammation and attenuate both oxidative stress and inflammation following an acute bout of physical activity [20]. Therefore chronic exercise training may be associated with an attenuation of airway inflammation in the postprandial period. Chronic exercise training also leads to an increased capacity to oxidize free radicals (i.e., an increased antioxidant status) [21]. A HFM leads to a transient increase in ROS, activating NF*κ*B [22]; however this increase may by lessened in active individuals due to an increased capacity to scavenge free radicals. It is possible that this may lead to a decrease in sensitivity to the HFM. Additionally, an acute bout of physical activity in the postprandial period is effective in decreasing PPL and proinflammatory cytokines [23]. A single bout of moderate-intensity exercise (~60% VO_2max⁡_) has been shown to decrease PPL in physically active subjects [24]. The reduction in PPL from aerobic exercise is beneficial from 16 hours prior to a HFM to 1.5 hours after HFM [25]. An acute bout of exercise also is marked by a transient increase in antioxidants that may protect the vascular endothelium after a high-fat meal [26]. These protective effects in the vascular endothelium may translate to the airway endothelium.

Therefore the primary aim of the study was to investigate whether an acute bout of moderate physical activity after a HFM would attenuate PPL as well as modulate markers of airway inflammation. We hypothesized that moderate intensity physical activity in the postprandial period in active individuals would attenuate the airway inflammatory response to a HFM compared to remaining inactive in the postprandial period.

## 2. Methods

### 2.1. Subjects

Seventeen college-aged participants (24.7 ± 5.9 years) took part in the study (11 M/6 F). All subjects were nonsmokers and were free of pulmonary, metabolic, or cardiovascular diseases determined via a medical history questionnaire. All subjects were active and exceeded physical activity guidelines (i.e., engaged in moderate-vigorous physical activity (MVPA) ≥ 150 minutes per week), determined via accelerometry. Written and verbal consent were obtained from all subjects. The study was approved by the Institutional Review Board Involving Human Subjects at Kansas State University and conformed to the Declaration of Helsinki.

### 2.2. Experimental Design

Subjects visited the laboratory two separate times, with one week in between trials. On the first visit, height, weight, and waist to hip ratio were assessed. Height was measured to the nearest 0.1 cm with a portable stadiometer (Invictus Plastics, Leicester, England), and body mass was measured to the nearest 0.1 kg with a digital scale (Pelstar LLC, Alsip, IL, USA). A dual-energy X-ray absorptiometry (DEXA) scan was performed to assess body composition (GE Lunar Prodigy, Madison, WI, USA). Pulmonary function tests (PFTs) were performed to ensure that subjects had normal pulmonary health (MIR Winspiro Pro, Waukesha, WI, USA). Subjects then completed a VO_2peak_ test on a treadmill to determine aerobic capacity, as well as the duration of submaximal exercise if they were randomized to the experimental (EX) group. Subjects then received Actigraph GT3X accelerometers to assess physical activity for the week (Actigraph, Pensacola, FL, USA).

Randomization occurred once subjects signed up for the study by a random number generator (IBM SPSS Statistics v22.0, IBM Corporation, Armonk, NY). The random number generator assigned either a (1) for control (CON) condition or a (2) for the exercise (EX) condition. In CON, subjects remained sedentary in the postprandial period while in the EX, subjects exercised at 60% of VO_2peak_ (a brisk walk) to expend half the calories they consumed from the HFM. The subjects began the bout of exercise 60 minutes after consuming the HFM. There were no dropouts after the subjects' initial assessment. Subjects picked up the prepackaged foods the night before the HFM and were asked to consume only the foods provided and to return any foods that were not consumed. Additionally, in the week after their initial assessment and prior to the HFM testing day, subjects kept a detailed 3-day food log.

**Figure 1:**
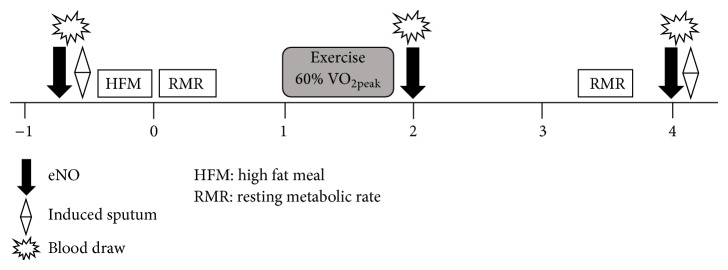
Experimental protocol for HFM testing day.

One week following their initial assessment, subjects visited the lab for their HFM testing session after a 12-hour fast and abstaining from caffeine or alcohol. They were also asked not to exercise for 24 hours prior to testing. Subjects first completed measurements of exhaled nitric oxide (eNO) followed by sputum induction. Afterwards, a fingerstick was performed to measure triglycerides, glucose, and cholesterol. Subjects then had 20 minutes to consume the HFM (described below). Immediately after the HFM subjects underwent resting metabolic rate measurements, which were repeated 200 minutes after HFM, blood draws were performed at 2 and 4 hours after HFM. Exhaled nitric oxide was assessed at 2 and 4 hours after HFM and sputum induction and processing were performed at baseline and 4 hours after HFM. The experimental protocol is depicted in the flow diagram (Figure 1).

### 2.3. Experimental Measures

#### 2.3.1. Dietary Recall Log

A 3-day food record was used to determine percent fat in the diet. Dietary intake was measured with a 3-day food record during the week between lab visits. Subjects were trained to accurately complete the dietary logs and diet was recorded on two weekdays and one weekend day. Dietary intake was entered and analyzed by a trained research assistant with Nutritionist Pro nutrient analysis software version 5.2.0 (Axxya Systems, Nutritionist Pro, Stafford, TX).

#### 2.3.2. Pulmonary Function Assessment

Pulmonary function tests were carried out during the first visit to the lab to assess pulmonary health. Subjects performed standard PFTs (peak expiratory flow (PEF), forced vital capacity (FVC), forced expiratory volume in 1 s (FEV_1_), and forced expiratory flow at 25–75% of vital capacity (FEF_25–75_)) a total of three times and within 10% of one another, and the maximal value was recorded in accordance with the American Thoracic Society Guidelines [27].

#### 2.3.3. Peak Aerobic Capacity

An incremental test on a treadmill was performed to assess peak oxygen consumption (Precor 932i). Heart rate was measured continuously using a (Polar Wear Link Coded) chest strap heart rate monitor. Metabolic and ventilatory data were assessed continuously through breath-by-breath analysis (Parvo Medics TrueOne 2400 Metabolic Cart, Sandy, UT). A modified Borg rating of perceived exertion (RPE) scale from 0 to 10 was used to assess perceived effort at the end of each 3-minute stage. The incremental protocol began after a four-minute warm-up at the subjects fastest 5 k pace on 2% incline. The speed was increased by 0.5 mph every two minutes and increased by 0.5 mph and 1% incline every two minutes past the third stage. Heart rate and VO_2_ were recorded at the end of each stage and upon completion of the test, and RPE was recorded 30 seconds prior to the end of each stage.

#### 2.3.4. High-Fat Meal

The HFM consisted of 10 kcal/kg of body weight (Jimmy Dean's Meat Lover's Breakfast bowl; 63% fat). Subjects were required to ingest the meal within 20 minutes of their first bite [15, 19]. The nutritional make-up of the meal was 460 calories per bowl, 33 grams of fat, 265 milligrams of cholesterol, 17 grams of total carbohydrate, and 24 grams of protein. Total kilocalories consumed ranged from 554.0 to 1113.0, while kilocalories from fat ranged from 349.0 to 701.2.

#### 2.3.5. Blood Sampling

Blood sampling was done at baseline prior to the HFM and at two hours and four hours after the HFM. A fingerstick was performed to measure triglycerides, glucose, and cholesterol. The fingerstick was performed and blood was collected in a capillary tube and analyzed using LDX cassettes (Cholestech LDX Analyzer, Alere San Diego Inc., San Diego, CA).

#### 2.3.6. Resting Metabolic Rate

Indirect calorimetry using a ventilated metabolic hood was used to assess resting metabolic rate (Parvo Medics TrueOne 2400 Metabolic Cart, Sandy, UT). Subjects were instructed to lie down with their head raised on a pillow for RMR measurements. They were instructed to remain still on the bed with the hood over their head for 30 minutes. An investigator stayed with the subject to ensure the blower for CO_2_ remained at 1%. The first five minutes and the last five minutes were not included in the data. All other data were recorded every five minutes during the testing session and averaged for calculation of macronutrient utilization, resting energy expenditure, and estimated energy expenditure during the testing procedure duration.

#### 2.3.7. Exhaled Nitric Oxide

Nitric oxide in exhaled breath was measured via chemiluminescence (Sievers Nitric Oxide Analyzer 280, Sievers Instruments Inc., Boulder, CO, USA). Subjects were asked to inhale to total lung capacity and steadily exhale at a constant flow rate for 6 seconds while data were recorded in real time. All tests were performed according to ATS Guidelines for measuring eNO [28]. The test was performed three times, and values within 5% of one another were averaged and included in analysis [15, 19].

#### 2.3.8. Sputum Induction

For sputum induction, hypertonic saline (5%) was administered for up to 30 minutes via an ultrasonic nebulizer (Omron Healthcare, Lake Forest, IL, USA). The aerodynamic diameter was 5 *μ*m and the output was 0.7 mL/min. Subjects were asked to rinse their mouth out carefully and try to expectorate sputum into a container every five minutes. The nebulizer was stopped after 30 minutes or earlier if enough sample was collected and a selective plug could be obtained [29].

#### 2.3.9. Sputum Processing and Analysis

All samples were processed within two hours of sputum induction. The quality, size, and number of plugs were recorded and treated with 0.1% dithiothreitol (DTT) in diluted water (4 times by weight) and phosphate-buffered saline (PBS) [30]. Cell viability was assessed by the trypan blue exclusion method and recorded from counts performed on the hematocytometer. The remaining filtrate was resuspended and three cytospins were made from every sample with 100–150 *μ*L at 500 rpm for 15 minutes. Slides were stained with Diff-Quick and mounted with Permount. Salivary contamination was assessed using methods of Pizzichini and colleagues [31]. In 20 subjects, 55% completed the sputum induction measurements at baseline and the 4-hour time point, and an additional six completed baseline measurements only and either were not able to expectorate at 4 hours or the sample was not countable. Sputum cell differentials were performed by a blinded investigator. Cell differentials were performed the same as validated methods from Telenga and colleagues [32].

#### 2.3.10. Statistical Analysis

Data analyses were conducted using IBM SPSS Statistics v22.0 (IBM Corporation, Armonk, NY). Pearson's 2-tailed correlations were used to determine associations between independent and dependent variables. All parametric assumptions were met and therefore appropriate parametric tests were used to determine differences between and within groups. Statistical comparisons between conditions (EX versus CON) at each time point were performed using a one-way analysis of variance (ANOVA). For blood analytes, a 2 (condition) × 3 (time) mixed factors ANOVA was performed, where condition was the between-subjects factor and time (baseline, 2 hours, and 4 hours) was the within-subjects factor. This was followed by post hoc Bonferroni tests to test pairwise comparisons. For metabolic and airway cell differentials, 2 (condition) × 2 (time) mixed factors ANCOVAs were conducted, with baseline inflammatory cell levels entered as covariates. For all analyses, the significance criterion was set at *P* < 0.05.

## 3. Results

### 3.1. Subject Characteristics

Subject characteristics and baseline pulmonary function measurements are shown in Table 1. Average age for the CON and EX was 22.4 ± 3.3 years and 27.3 ± 7.3 years, respectively, and average body mass was 79.3 ± 16.4 kg for CON and 70.7 ± 10.2 kg for EX. There were no significant differences between the CON and EX subject characteristics at baseline. Subjects had PFT values within normal ranges and percent predicted values at baseline according to ATS Guidelines [30]. Average FVC for the CON and EX was 6.0 ± 1.9 L and 4.7 ± 1.3 L, respectively.

### 3.2. Blood Analytes

**Figure 2:**
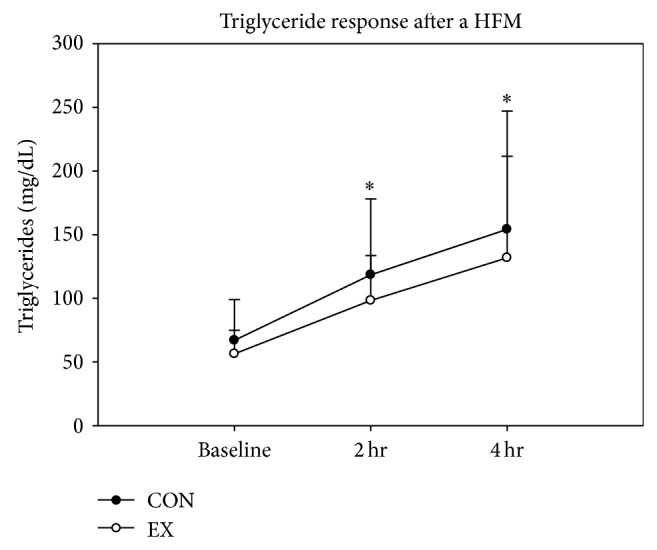
Triglyceride response after HFM. Black circles indicate CON and white circles indicate EX. There was a significant increase (∗) in triglycerides at two hours and four hours after HFM in both CON and EX. However, there was no significant difference between CON and EX (*P* > 0.05).

Mean values for blood analytes are expressed in Table 2.  Figure 2 displays the increase in triglycerides in both CON and EX at two hours and four hours after HFM. Triglycerides doubled in the postprandial period (*P* < 0.05) but did not differ between CON and EX. Total cholesterol (TC) significantly increased in EX at two hours when compared to CON (*P* = 0.001), but there was no significant main effect of time when comparing baseline and two and four hours after HFM (*P* > 0.05), although the interaction of TC and condition was significant (*P* = 0.044). HDL did not significantly change over time; however it significantly decreased in CON from baseline to two hours compared to EX (*P* = 0.025). The interaction of HDL and condition was not significant (*P* = 0.064). LDL significantly decreased from baseline to four hours in CON (*P* < 0.05) and not in EX (*P* = 0.18) and was significantly lower in CON at two hours and four hours versus EX (*P* = 0.047). There was a significant interaction between LDL and condition as a quadratic function (*P* = 0.047). Glucose did not significantly increase at any time point or by condition (*P* > 0.05); however the interaction of glucose and condition was significant as a quadratic function, where EX was higher than CON at two hours and decreased at four hours (*P* < 0.001). The ratio of total cholesterol to HDL (TC/HDL) was significantly increased over time (*P* = 0.026), but there was no difference between CON and EX and no interaction effect.

### 3.3. Metabolic Data

Mean values for metabolic data are shown in Table 3. Respiratory exchange ratio (RER), resting energy expenditure (REE), and kilocalories of carbohydrate utilized significantly increased at 200 minutes after HFM as a main effect of time (*P* < 0.05), but not by condition (*P* > 0.05). There were not significant outcome measures by condition alone (*P* > 0.05); however the interaction between time and condition was significant in percent of carbohydrate (*P* = 0.019), percent of fat (*P* = 0.019), kilocalories of fat (*P* = 0.025), and kilocalories of carbohydrate (*P* = 0.005) utilized.

### 3.4. Airway Inflammation

**Figure 3:**
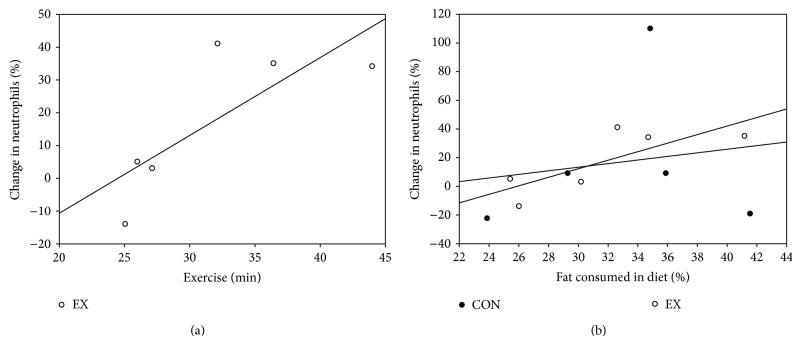
(a) displays the percent change in neutrophils present at 4 hours which was significantly correlated with minutes of walking in the exercise bout (*r* = 0.879, *P* = 0.050). (b) displays the percent of kilocalories of fat in the diet which is significantly correlated with the percentage increase of neutrophils in EX (*r* = 0.826, *P* = 0.043), but not in CON.

Figure 3(a) illustrates that the percentage change in neutrophils from baseline to 4 hours in the EX was associated with minutes of walking in the exercise bout (*r* = 0.879, *P* = 0.050). The percent of fat intake determined via the food log was positively associated with the percent change in neutrophils from baseline to four hours in EX (*r* = 0.826, *P* = 0.043) and was not significant in the CON (Figure 3(b)). The percent change in neutrophils present at 4 hours was significantly correlated with the amount of kcals consumed per kilogram of body weight in the CON (*r* = 0.954, *P* = 0.012) and not in EX.

**Figure 4:**
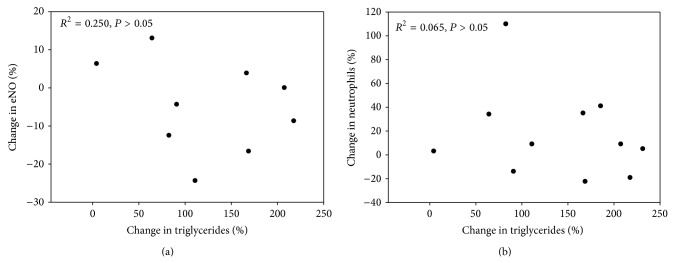
Percent change in triglycerides and airway inflammation four hours after HFM. Percentage change in triglycerides and percentage change in exhaled nitric oxide (ppb) is depicted in (a). There was no significant association between the percentage increase in triglycerides and the percentage increase in eNO (*R* ^2^ = 0.250, *P* > 0.05). Percentage change in triglycerides as well as percentage change in pulmonary neutrophils is depicted in (b). There was no significant association between the percentage increase in triglycerides and the percentage increase in pulmonary neutrophils (*R* ^2^ = 0.065, *P* > 0.05).

Airway inflammation data are displayed in Table 4. There was a significant increase in neutrophils and eosinophils in the postprandial period as a main effect of time (*P* < 0.05) but was not different between CON and EX (*P* > 0.05). There was no significant interaction in any inflammatory cells with condition. There was no significant linear relationship for eNO across these assessments; however, there was a significant quadratic relationship, *F*(1,10) = 5.70, *P* < 0.05, *η* ^2^ = 0.36, such that, averaging over the EX and CON condition, the eNO tends to be lower in the first and last trials, but higher at the 2-hour time point. There were no significant differences in eNO between the CON and EX condition. The percent increases in eNO and sputum neutrophils were not associated with the percent change in triglycerides (*P* > 0.05) (Figures 3(a) and 3(b), resp.). There were no significant associations between the increases in eNO with the presence of eosinophils or neutrophils four hours after HFM. Additionally, there were no significant associations between the percentage increase in triglycerides with percentage increase in eNO or neutrophils (Figures 4(a) and 4(b), resp.)

## 4. Discussion

### 4.1. Major Findings

The current study suggests that a moderate intensity exercise bout performed one hour after a HFM is not effective in reducing PPL and subsequent airway inflammation in active subjects. Both the CON and EX groups showed significantly increased PPL after a HFM, and there were no statistically significant differences between the groups. However, pulmonary eosinophils, neutrophils, and exhaled nitric oxide did increase after HFM. The increase in the presence of inflammatory cells in both the CON and EX groups is an unexpected finding. The increases in eNO, neutrophils, and eosinophils in both the CON and EX groups were not associated with one another and therefore will be a highlighted topic of this discussion.

### 4.2. Physical Activity Level and Postprandial Airway Inflammation

Our study is the first to account for physical activity status in the postprandial airway response to a HFM. Our findings indicate that subjects with high levels of physical activity (≥150 minutes/week) have similar airway inflammatory responses following a HFM as compared to previous research in subjects with lower physical activity levels [17]. Previous research has shown that chronic exercise training increases antioxidant status and the ability to scavenge reactive oxygen species (ROS) [33]. ROS and oxidative stress occur after a single HFM due to activation of NF*κ*B by endotoxin (lipopolysaccharide (LPS)) in both animals and humans [34]. However, chronic exercise training reduces NF*κ*B activation and leads to decreased TLR4 mRNA expression [35]. This could potentially reduce neutrophil influx and the inflammatory burden, although it has never been investigated in the airways. We did not measure antioxidant status directly; however, it is possible that the subjects' activity levels may still have attenuated airway inflammation if compared to less active subjects or overweight and obese individuals. A high-fat meal causes significantly greater NF*κ*B activation and prolonged ROS production in obese subjects than in healthy weight controls [22]. Future research should be conducted to determine how chronic physical activity modulates the lung immune response. Additionally, the exercise bout in the present study did not lower PPL. Active individuals have increased lipid turnover and clearance and therefore lower area under the curve (AUC) and peak triglycerides [36]. Therefore it is probable that the inflammatory burden of a HFM would be much greater systemically and in the airways in physically inactive or overweight/obese individuals.

### 4.3. Triglycerides and Postprandial Airway Inflammation

Existing studies have reported a correlation between blood triglycerides and exhaled nitric oxide [15, 19], which was not found in the present study. Both of these studies showed that eNO and TG at baseline and two hours after HFM were associated; however the postprandial increase in TG was not associated with the eNO response to the HFM in either study. Similarly in the present study, the increase in eNO at any time point was not associated with triglycerides. However there may be a greater magnitude in the airway inflammatory response in subjects who display greater PPL (e.g., sedentary or overweight/obese subjects). Increased PPL increases the risk for developing both asthma and obesity [37], so it is important to determine the magnitude of the inflammatory response after a HFM in these subjects, as well as how chronic diet and exercise may also moderate airway inflammation in the postprandial period.

### 4.4. Explanation for Increases in Airway Inflammation after HFM

Previous research has reported that dietary fat activates innate immune mechanisms in the airway, as described previously (see Section 1). Recent animal studies have found that high-fat challenges recruit inflammatory cells [38], but only one human study has found increased neutrophils in the postprandial period in healthy control subjects [17]. Therefore our results are in agreement with only the other human study, to our knowledge, performed in nonasthmatic subjects that found an increased presence of pulmonary neutrophils after a HFM. Interestingly, our research study is the first to find an increase in pulmonary eosinophils after a HFM in healthy subjects, although due to the small sample and high standard deviations, this is unlikely to be clinically significant in healthy individuals. These findings may be particularly relevant to obese or healthy weight asthmatics who may experience worsened symptoms when consuming a chronically high fat diet associated with presence of both eosinophilic and neutrophilic airway inflammations.

Several other studies have reported a significant linear increase in eNO from baseline to two hours after HFM [15, 19]. Conflicting studies have found there was not a significant increase in eNO measured at baseline and four hours after HFM [17]. This is the first study to measure eNO at two and four hours after HFM, which may elucidate the conflicting results in the previous studies. In the present study, exhaled nitric oxide significantly increased from baseline to two hours after HFM as a quadratic function, rather than a linear function as previously described. Therefore there was no statistical significance from baseline to two hours, but there was significance when considering all three time points nonlinearly. It appears that the eNO does return near baseline values four hours after HFM. It is clearly important to consider multiple time-points as a way to interpret the time course in which eNO may be associated with both systemic markers of inflammation and the inflammatory cell recruitment in the airway.

### 4.5. Potential Mechanisms

A novel finding in the present study was an increase in eNO as a quadratic function as well as increased airway inflammatory cells four hours after the HFM, yet there were no significant differences between the CON and EX conditions. However since changes in eNO, eosinophils, and neutrophils were not associated with one another, it is possible that the airway inflammation occurred via different mechanisms. Exhaled nitric oxide was not associated with sputum neutrophils or eosinophils, so we must consider other sources that may modulate the presence of inflammatory cells independent of one another. Although studies report active individuals have an increased capacity to oxidize free radicals [35], which would attenuate the transient increase in ROS after HFM, studies have also reported ROS increase immediately after exercise [39]. Therefore it is possible that the increase in ROS from exercise led to subsequent neutrophil influx and activation. Since increases in presence of neutrophils were significantly associated with minutes of walking in EX, it is possible that the increased airway inflammation was the result of the acute exercise bout, although the bout of exercise in the present study was a lower exercise intensity than in the existing literature [40, 41]. Increases in eNO have been found with exercise [42], although this is not a consistent finding [43]. The existing literature investigating the inflammatory burden of various intensities of exercise is scarce and needs to be explored in greater detail.

### 4.6. Important Considerations in Modulation of Airway Inflammation

Along with decreased physical activity, a chronic high fat diet has been linked to decreased antioxidant status [44]. In the present study, there was a significant correlation between chronic dietary fat intake and percent change in neutrophils in the EX group only, which may be an important moderating variable to consider in future studies. Wood and colleagues determined dietary modification modulates airway inflammation in an acute high fat challenge, but no studies have reported chronic diet and the airway inflammatory response. Therefore it is possible that chronic diet may play a moderating role in the airway inflammatory response to both postprandial airway inflammation and exercising in the postprandial period. This concept may have clinical utility when recommending exercise interventions and nutritional guidance to overweight and obese subjects. Studies have reported higher saturated fatty acid intake in the normal diet of overweight and obese subjects [45]. If chronic dietary fat intake may exacerbate airway inflammation during exercise, it may be important to consider modulating diet as a means to reduce the response to exercise, which may reduce asthma symptoms severity during exercise [46].

### 4.7. Future Directions

This study provides interesting insight into the innate immune mechanisms that may cause airway inflammation after a HFM. Research thus far has primarily investigated airway inflammation after HFM after modulating the composition of fatty acids in an acute high-fat challenge. It is necessary to develop other strategies to reduce the postprandial inflammatory burden on the airways that is present in asthmatics. This may be beneficial in improving clinical outcomes and therefore warrant further investigations. No previous studies have accounted for physical activity, which could reduce PPL and subsequent airway inflammation. Antioxidant status may play a role in modulation of airway inflammation in the postprandial period via activation of NF*κ*B. Therefore an exercise intervention to determine if this response is modifiable is important. Additionally, future research studies must focus on determining therapeutic methods to attenuate the airway inflammatory response, specifically in high risk populations or people already diagnosed with asthma.

## Figures and Tables

**Table 1:** Subjects' characteristics.

	CON (7 M/2 F)	Range	EX (4 M/4 F)	Range
	Value ± SD		Value ± SD	
Age (years)	22.4 ± 3.3	19–30	27.3 ± 7.3	19–38
Height (cm)	177.0 ± 9.6	165.4–193.5	170.4 ± 7.8	162.6–183.1
Weight (kg)	79.3 ± 16.4	62.3–111.3	70.7 ± 10.2	55.5–87.5
Body mass index (BMI) (kg/m^2^)	25.1 ± 2.9	20.9–29.7	24.4 ± 3.1	19.5–27.9
Body fat (%)	16.1 ± 6.8	5.4–24.3	23.5 ± 11.1	8.5–42.2
Waist circumference (cm)	84.8 ± 5.1	77.5–91.3	85.5 ± 6.3	75.7–95.1
Systolic (mmHg)	120.6 ± 14.7	98–141	114.0 ± 6.4	108.5–126.5
Diastolic (mmHg)	65.1 ± 9.4	50–79	66.8 ± 7.5	60.5–82.0
VO_2_ (L/min)	4.3 ± 0.9	3.3–5.6	3.9 ± 1.0	2.9–5.6
VO_2peak_ (mL/kg/min)	54.2 ± 4.7	47.2–63.1	54.9 ± 11.1	39.2–68.9

Baseline pulmonary function tests	CON (7 M/2 F)	Percent of predicted	EX (4 M/4 F)	Percent of predicted
	Value ± SD		Value ± SD	

PEF (L/s)	9.4 ± 3.02	109.1 ± 22.1	8.3 ± 2.3	104.3 ± 9.5
FVC (L)	6.0 ± 1.9	121.1 ± 21.8	4.7 ± 1.3	108 ± 11.6
FEV_1_ (L)	5.1 ± 1.5	5.1 ± 1.5	4.2 ± 1.2	111.4 ± 15.2
FEV_1_/FVC	86.7 ± 7.3	86.7 ± 7.3	87.8 ± 2.0	105.8 ± 3.7
FEF_25–75%_ (L/s)	5.4 ± 1.5	112.1 ± 23.1	4.8 ± 1.2	108.3 ± 18.9

Values are expressed as mean ± SD. There were no significant differences between CON and EX at baseline, *P* > 0.05.

PEF: peak expiratory flow; FVC: forced vital capacity; FEV_1_: forced expiratory volume in 1 s; FEV_1_/FVC (%): ratio of forced expiratory volume in 1 s to forced vital capacity; FEF_25–75_: forced expiratory flow between 25 and 75%.

**Table 2:** Blood analyte values at baseline and 2 hours and 4 hours after HFM.

	Baseline		2 hr		4 hr	
	CON	EX	CON	EX	CON	EX
	Value ± SD	Value ± SD	Value ± SD	Value ± SD	Value ± SD	Value ± SD
*n* =	9	8	9	8	9	8
TG (mg dL^−1^)	67.0 ± 32.0	56.4 ± 18.5	118.4 ± 59.7^*^	98.3 ± 35.3^*^	154.2 ± 92.9^*^	131.9 ± 79.7^*^
TC (mg dL^−1^)	143.6 ± 16.3	145.0 ± 21.4	135.0 ± 13.3	154.5 ± 17.2^‡^	149.2 ± 25.7	147.5 ± 14.9
HDL (mg dL^−1^)	50.4 ± 12.9	56.8 ± 11.6	45.1 ± 8.4	59.1 ± 8.3^‡^	43.8 ± 8.5	54.9 ± 7.9
LDL (mg dL^−1^)	80.3 ± 12.6	75.3 ± 28.6	64.9 ± 19.2^*^	74.7 ± 17.5^‡^	62.8 ± 19.9^*^	66.1 ± 14.9^‡^
Glucose (mg dL^−1^)	84.4 ± 6.4	82.6 ± 13.7	76.2 ± 6.5	92.1 ± 10.6^‡^	86.1 ± 4.7	86.6 ± 7.7
TC/HDL	3.0 ± 0.7	2.6 ± 0.5	3.1 ± 0.7^*^	2.7 ± 0.4	3.3 ± 0.7^*^	2.7 ± 0.6

Blood analyte data at baseline and 2 hr and 4 hr after HFM in CON versus EX. Values are expressed as mean ± SD.

^*^Significantly different from baseline (*P* < 0.05).

^‡^Significantly different from CON (*P* < 0.05).

TG: triglycerides; TC: total cholesterol; HDL: high-density lipoprotein; LDL: low-density

lipoprotein; glucose; TC/HDL: ratio of total cholesterol to high-density lipoprotein.

**Table 3:** Metabolic data at baseline and 200 minutes after HFM.

	Baseline		200 minutes	
	CON (*n* = 8)	EX (*n* = 8)	CON (*n* = 9)	EX (*n* = 8)
	Value ± SD	Value ± SD	Value ± SD	Value ± SD
Respiratory exchange ratio (RER)	0.8 ± 0.04	0.8 ± 0.05	0.8 ± 0.04^*^	0.8 ± 0.03
Resting energy expenditure (REE)	1909.5 ± 566.8	1741.9 ± 397.1	2177.7 ± 510.4^*^	1873.0 ± 452.6
Carbohydrates utilized (%)	31.4 ± 12.7	32.1 ± 16.1	44.8 ± 17.8	27 ± 9.5
Fat utilized (%)	68.1 ± 12.6	67.3 ± 15.9	54.7 ± 14.7	72.1^‡^ ± 9.6
Carbohydrate (kcal/day)	639.8 ± 451.8	581.8 ± 361.3	1027.2 ± 536.2^*^	517.3 ± 262.5^*^
Fat (kcal/day)	1257.6 ± 308.2	1148.9 ± 319.3	1136.8 ± 238.6	1343.3^‡^ ± 343.9^*^

Metabolic data at baseline and 200 minutes after the HFM. Values are expressed as mean ± SD. There is one missing piece of data at baseline in CON condition. Asterisks indicate being significantly different at 200 minutes from baseline (*P* < 0.05). ^‡^Significantly different from CON (*P* < 0.05).

**Table 4:** Airway inflammatory cell differentials at baseline and 4 hours after HFM.

	Baseline		4 hr	
	CON (*n* = 9)	EX (*n* = 8)	CON (*n* = 5)	EX (*n* = 6)
	Mean ± SD	Mean ± SD	Mean ± SD	Mean ± SD
Total cell counts (×10^6^/L)	Range: 2.0–2.6		Range: 2.0–2.4	
Percentage of macrophages	28.9 ± 11.8	38.1 ± 15.1	26.9 ± 13.4	28.7 ± 9.3
Percentage of neutrophils	61.3 ± 15.8	53.3 ± 16.8	64.8 ± 10.4^*^	64.5 ± 15.0^*^
Percentage of eosinophils	0.3 ± 0.4	0.5 ± 0.5	0.5 ± 0.8^*^	0.5 ± 0.7^*^
Percentage of basophils	1.3 ± 1.4	0.9 ± 0.8	0.4 ± 0.5	0.8 ± 0.9
Percentage of lymphocytes	8.6 ± 4.7	7.7 ± 5.7	7.6 ± 3.7	7.6 ± 4.7

Airway inflammatory cell differentials in CON and EX at baseline and 4 hr after HFM. Values are expressed as mean ± SD. Asterisks indicate that there was a significant increase in percentage of pulmonary neutrophils and eosinophils from baseline to 4 hours after HFM (*P* < 0.05). There were no significant differences between CON and EX (*P* > 0.05).
